# Knowledge of malaria influences the use of insecticide treated nets but not intermittent presumptive treatment by pregnant women in Tanzania

**DOI:** 10.1186/1475-2875-3-42

**Published:** 2004-11-12

**Authors:** Rhoida Y Nganda, Chris Drakeley, Hugh Reyburn, Tanya Marchant

**Affiliations:** 1 Kilimanjaro Christian Medical Centre, PO Box 3010, Moshi, Tanzania; 2 Joint Malaria Programme, PO Box 2228, Moshi, Tanzania; 3 London School of Hygiene and Tropical Medicine, Keppel Street, London WC1E7HT, UK

## Abstract

**Background:**

To reduce the intolerable burden of malaria in pregnancy, the Ministry of Health in Tanzania has recently adopted a policy of intermittent presumptive treatment for pregnant women using sulphadoxine-pyrimethamine (IPTp-SP). In addition, there is strong national commitment to increase distribution of insecticide treated nets (ITNs) among pregnant women. This study explores the determinants of uptake for both ITNs and IPTp-SP by pregnant women and the role that individual knowledge and socio-economic status has to play for each.

**Methods:**

293 women were recruited post-partum at Kibaha District Hospital on the East African coast. The haemoglobin level of each woman was measured and a questionnaire administered.

**Results:**

Use of both interventions was associated with a reduced risk of severe anaemia (Hb<8 g/dL) compared to women who had used neither intervention (OR 0.31, 95% CI 0.14–0.67). In a logistic regression model it was found that attendance at MCH health education sessions was the only factor that predicted IPTp-SP use (OR 1.8, 95% CI 1.1–2.9) while high knowledge of malaria predicted use of ITNs (OR 2.3, 95% CI 1.1–4.9).

**Conclusion:**

Individual knowledge of malaria was an important factor for ITN uptake, but not for IPTp-SP use, which was reliant on delivery of information by MCH systems. When both these interventions were used, severe anaemia postpartum was reduced by 69% compared to use of neither, thus providing evidence of effectiveness of these interventions when used in combination.

## Background

*Plasmodium falciparum *malaria in pregnancy poses a substantial risk to pregnant women and their offspring: it has been estimated that malaria in pregnancy is the primary cause of up to 10,000 maternal anaemia-related deaths in sub-Saharan Africa annually [1]. Further, malaria in pregnancy increases the risk of an infant being born with low birth weight (LBW) and is responsible for up to 35% of preventable LBW in malaria-endemic areas[2].

Over the last decade a body of evidence has accumulated which supports the use of both ITNs and IPTp-SP to reduce the adverse effects of malaria during pregnancy [1,3,4] and both these interventions are currently recommended by WHO [5]. In Tanzania it is now national policy to offer IPT-p with sulfadoxine-pyrimethamine (SP) for every pregnant woman attending Maternal and Child Health services (MCH). There is strong commitment in Tanzania to achieve the targets agreed on by African countries at the Abuja Conference of 60% coverage of ITNs for pregnant women by 2005 using social marketing strategies and a national voucher scheme [6] to improve access for pregnant women and their children. However, there are currently no reliable data in Tanzania on current levels of use of these interventions.

Evidence suggests that malaria treatment choices are affected by knowledge of the problem [7,8]. The success in implementing preventive interventions amongst pregnant women in Tanzania is thus likely to be determined in part by awareness of malaria and the strategies available to prevent it. This study set out to explore the determinants of uptake for both ITNs and IPTp-SP by pregnant women, with particular regard to knowledge of malaria and socio-economic status, and to estimate the impact that reported use of either of these interventions had on the prevalence of severe anaemia in the post-partum period.

## Methods

### Setting

This research was carried out at Kibaha District Hospital in the Coastal Region of Tanzania, an area with moderate to high malaria transmission. The area is predominantly rural, supported by a mixture of subsistence and cash crop farming, with a peri-urban belt along the main Tanzania/Zambia highway which crosses the district.

### Study participants

All post-natal women who had delivered their baby in the hospital during April/May 2003, but had not been admitted to hospital during the pregnancy, were eligible for inclusion in the study.

### Study tools

From studies elsewhere [9] it was estimated that a samples size of 293 women would give sufficient power to show a difference in uptake of malaria interventions by knowledge of malaria. On each working day of the study the first 13 women to come out of the labour ward and who gave informed consent to participate were registered and interviewed. Haemoglobin level was determined at the time of interview using a portable β-haemoglobin photometer (HemoCue^©^, HemoCue AB, Ängelholm, Sweden). Women with Hb<11 g/dL were referred within the hospital system.

### Definitions

#### Knowledge of malaria score (KoM)

Participants were assigned a 'knowledge of malaria score' (KoM) according to their responses to a series of seven closed questions (Table 1).

**Table 1:** Components of the knowledge score

Malaria in pregnancy statement:		Agreement	
		n/293	%
Risk of infection	Increases in pregnancy	270	92
Consequences	Low birth weight	81	28
	Pregnancy Loss	112	38
	Maternal anaemia	150	51
Best Interventions*	ITN	266	91
	SP	115	39
Transmission	Mosquitoes alone	102	35

*Women asked to state up to two malaria interventions

#### ITN users

Women who reported that during this pregnancy they had normally (i.e. >75% of the time) slept under a bednet which had been impregnated within the previous six months.

#### IPTp-SP users

Use of intermittent presumptive treatment of malaria in pregnancy with sulfadoxine-pyrimethamine.

#### Severe anaemia

Severe anaemia post-partum was defined as Hb<8 g/dL.

### Data analysis

Data were entered twice in Epi-Info version 6.04 and analysed in Stata version 7 (Stata Corporation, Texas USA). Evidence of an association was sought between the variables listed in Table 1 and the study outcomes (reported use of interventions or severe anaemia defined as Hb<8 g/dl). Variables found to have an association with each outcome (by χ^2 ^P-value <0.10 or Mantel-Haenszel estimate of the rate ratio, P-value <0.10) were further analysed using multiple logistic regression. Significance in the multi-variate models was defined by a likelihood-ratio test (LRT) P-value <0.05.

## Results

A completed questionnaire and measurement of haemoglobin level were available for 293 post-partum women whose characteristics are shown in Table 1. The group was predominantly made up of married women in the 20–29 years age-group, with primary level education only. Both urban and rural residents were represented. No women refused to participate.

### Levels of knowledge

The KoM assessment consisted of four parts: knowledge of risk, consequences of risk, transmission and prevention, with seven questions in total (Table 1). Each question contributed one point to the overall KoM score (i.e. range of possible scores of 0–7) and overall the mean score was 3.7 (median 4). The KoM score was stratified into three groups: score 0–2 representing low (29% of respondents), score 3–4 representing median (38%) and score 5–7 representing high levels of knowledge (33% of respondents).

The lowest knowledge scores related to the impact of maternal malaria on the health of the foetus; only 28% (81/293) recognized low birth weight and 38% (112/293) pregnancy loss as a potential consequence of maternal malaria. There was also some confusion over the mode of transmission of malaria: 95% (279/293) agreed that mosquitoes could transmit malaria but only 35% thought that mosquitoes alone were responsible (excepting blood transfusion).

Having high KoM was strongly associated with education level (χ^2 ^for linear trend 50.03, p < 0.0001). Because of colinearity between education level and high KoM, education was excluded from the logistic regression model used to identify determinants of high KoM. However, models for primary and secondary education respectively showed the same socio-economic effects as the data presented in Table 3. In the logistic regression model, only ages above teenage (LRT 9.8, p = 0.007), ownership of a radio (LRT 6.0, p = 0.01), ownership of a bicycle (LRT 4.8, p = 0.02) and citing the MCH rather than the community as the most important source of health information (LRT 7.5, p = 0.02) were significantly associated with a high KoM compared to low/median KoM.

**Table 3:** Unadjusted and adjusted OR's for characteristics of women with high knowledge of malaria^1^.

		N	Unadjusted OR	Confidence Interval	Adjusted OR	Confidence Interval	LRT	P
Age	15–19	49	1.0	-	1.0	-		
	20–29	185	3.3	1.4–7.8	3.2	1.2–8.4		
	30+	59	4.4	1.7–11.4	4.6	1.6–13.3	9.8	<0.01
Radio	No	29	1.0	-	1.0	-		
	Yes	264	7.7	1.7–33.1	5.1	1.1–24.0	6.0	0.01
Bicycle	No	69	1.0	-	1.0	-		
	Yes	224	2.3	1.2–4.5	2.1	1.0–4.3	4.8	0.02
Source of health information	Community	36	1.0	-	1.0	-		
	MCH	83	5.8	11.8–18.0	4.3	1.3–14.0		
	Media	174	4.1	1.3–12.1	2.6	0.8–8.1	7.5	0.02

^1^All women with high malaria knowledge had formal schooling

### Use of IPT-p or ITN

48% (141/293) of women were ITN users, 57% (166/293) had received IPTp-SP once and 12% (34/293) IPTp-SP twice. A multi-variate analysis of factors influencing the uptake of these two interventions is shown in Table 4. After adjustment, increasing age (LRT 10.3, P = < 0.01), owning a radio (LRT 4.0, P = 0.04) and having a high KoM, compared to a low/median KoM score (LRT 7.3, P = 0.02), were the only factors that independently predicted use of an ITN in pregnancy (table 4). By contrast, only a history of having received health education during the pregnancy significantly predicted uptake of any dose of IPTp-SP (LRT 5.6 p = 0.01).

**Table 4:** Factors associated with uptake of insecticide treated nets (ITNs) during pregnancy and at least one dose of sulphadoxine-pyrimethamine as part of intermittent presumptive treatment (IPTp-SP)

		ITN users					
	N	Unadjusted OR	95% Confidence Interval	Adjusted OR	95% Confidence Interval	LRT	P
Health Education							
No	160						
Yes	133						
Age							
15–19	49	1.0	-	1.0	-		
20–29	185	3.2	1.6–6.1	2.5	1.3–5.0		
30+	59	4.2	1.8–10.3	3.8	1.5–9.4	10.3	<0.01
Knowledge							
Low	86	1.0	-	1.0	-		
Medium	110	1.3	0.7–2.3	1.0	0.5–1.8		
High	97	3.6	1.7–7.1	2.3	1.1–4.9	7.3	0.02
Radio							
No	29	1.0	-	1.0	-		
Yes	264	2.9	1.3–6.3	2.3	1.0–5.5	4.0	0.04
		Used IPTp-SP					
		Unadjusted OR	95% Confidence Interval	Adjusted OR	95% Confidence Interval	LRT	p
Health Education							
No	160	1.0	-	1.0	-		
Yes	133	1.8	1.1–2.9	1.8	1.1–2.8	5.6	0.01
Age							
15–19	49	1.0	-	1.0	-		
20–29	185	0.8	0.4–1.6	0.8	0.4–1.6		
30+	59	0.9	0.4–2.0	0.7	0.3–1.7	0.4	0.8
Knowledge							
Low	86	1.0	-	1.0	-		
Medium	110	1.7	1.0–3.0	1.7	1.0–3.1		
High	97	1.6	0.9–2.9	1.6	0.8–2.9	3.8	0.14
Radio							
No	29						
Yes	264						

41% (120/293) of women had used both interventions. Women with a median KoM were twice as likely (OR 2.1 (95% CI 1.1–4.0) and women with a high KoM three times more likely (OR 3.2 (95% CI 1.7–6.0) to have used both IPT-p and an ITN than women with low KoM scores.

### Timing of first MCH attendance

26% (76/293) of women had first attended the MCH during the first trimester, 65% (192/293) during the second trimester and 9% (25/293) during the third trimester of pregnancy. Predictably the median number of visits to MCH by women was correlated with the trimester of first visit: first trimester – median of 7.5 visits overall (mean 6.9 (s.d.2.2), second trimester – median of 5 visits overall (mean 5.4 (s.d.1.8) & third trimester – median of 2 visits overall (mean 2.4 (s.d.0.8). Women attending MCH for the first time during their first trimester were more than twice as likely to have attended health education sessions as women attending for the first time in their third trimester (χ^2 ^test for trend 5.1, p = 0.02).

### Risk factors for severe anaemia

Overall, 27% (80/293) of study participants had Hb<8 g/dL immediately post-partum. In the regression model, three factors were found to independently predict the risk of having severe anaemia in the post-partum period (Table 5). Firstly, with women using either an ITN or IPTp-SP alone, there was a reduced risk of severe anaemia, but it was not statistically significant (OR 0.77 (CI 0.36–1.65 and OR 0.70 (CI 0.34–1.41 respectively). However, the use of an ITN in conjunction with IPTp-SP was associated with a significant reduction in risk of being severely anaemic post partum compared to women not using any intervention (OR 0.31 (CI 0.14–0.67) LRT 10.1 p = 0.01). Secondly, having had any level of education (as compared to none) and, thirdly, attendance at the MCH clinic during the first rather than third trimester of pregnancy were both associated with a reduced risk of severe anaemia (OR 0.41, 95% CI 0.17–0.98) LRT 4.5 p = 0.03 and OR 0.31, 95%CI 0.11–0.83, LRT 5.3 p = 0.05 respectively).

**Table 5:** Risk factors for severe anaemia post partum.

	% with severe anaemia	Unadjusted OR	95% confidence interval	Adjusted OR	95% confidence interval	LRT	P
Formal education							
No	29	1.00	-	1.00	-		
Yes	16	0.45	0.19–1.07	0.41	0.17–0.98	4.5	0.03
Time of first ANC use							
3^rd ^trimester	48	1.00	-	1.00	-		
2^nd ^trimester	26	0.39	0.16–0.91	0.41	0.17–1.0		
1^st ^trimester	22	0.31	0.12–0.80	0.31	0.11–0.83	5.3	0.05
Use of malaria intervention							
None	37	1.00	-	1.00	-		
ITN only	32	0.82	0.39–1.71	0.77	0.36–1.65		
IPTp-SP only	27	0.64	0.32–1.28	0.70	0.34–1.41		
ITN + IPTp-SP	16	0.34	0.16–0.72	0.31	0.14–0.67	10.1	0.01

## Discussion

The key finding was that while knowledge, wealth and age were all found to be independently predictive of ITN use, only participation in health education was associated with use of IPTp-SP. That predictors of uptake for each of the two interventions were different was an interesting and unexpected finding. The data indicate that, while ITN use is driven by both access (i.e. wealth) as well as knowledge, use of IPTp is determined by its being offered in an MCH clinic. This is a positive finding that suggests the need to prioritise strategies for maximising early attendance to boost IPTp-SP uptake.

This finding is in contrast to research from Kenya which showed that uptake of IPTp-SP increased with higher levels of formal education [9]. The National Malaria Control Programme of Tanzania does not currently have any data on IPTp-SP uptake nationally, although it is planned for integration into routine surveillance next year. It is likely that distribution systems for this, as other clinic based interventions, will need more attention. It is possible that the new MCH clinic based voucher system for ITN will have an added benefit by increasing early attendance and therefore uptake of IPTp-SP.

These findings on knowledge and awareness suggest that the increased risk posed to pregnant women by malaria was almost universally recognized, but that knowledge of the health impact of that risk – especially to the health of the foetus – was very low. Over 90% of women thought that ITNs were a good intervention against malaria in pregnancy, but less than half thought the same about IPTp-SP. Knowledge of malaria in pregnancy was strongly associated with use of a combination of both ITN and IPTp. Although this was a small observational study where it was not possible to control for all likely confounding variables, there is evidence that the use of a combination of ITN use and IPT-p provides additive protection against severe anaemia, and that this effect is not confined to trial conditions. Use of an ITN or IPT-p alone was associated with a 23% and 30% reduction in the risk of severe anaemia post partum respectively, similar to estimates from other controlled trials [10,11] The data suggest that MCH services are effective when used optimally. Women who accessed MCH services in their first trimester of pregnancy had a significantly lower risk of severe anaemia post-partum compared to women who first presented at MCH during their third trimester. Women attending MCH earlier were more likely to attend health education sessions, and women who attended health education sessions more likely to use IPTp-SP than women who did not attend. Women with high KoM were most likely to cite the MCH as their most important source of health information.

It must be noted that by recruiting only women who had uncomplicated pregnancies and who delivered in hospital this study had a selection bias towards the healthier and possibly wealthy members of the community. However, women from a considerable range of socio-economic situations are represented (no education vs. further education; mobile phone owners vs. not owning a radio) which probably reflects the rural/peri-urban catchment area of Kibaha and other newly urbanised areas. The data has shown that many of the factors indicative of relative wealth in a poor community were associated with increasing levels of knowledge – and that knowledge was positively associated with multiple intervention uptake and improved health outcome.

The importance of access to resources has been illustrated previously for both preventative interventions and treatment [12]. At the time of this study, IPTp-SP was offered free of charge to pregnant women via the antenatal clinics in Tanzania. There is social marketing of bednets (price approx $5) at the national level but not targeted at specific high risk groups. The newly initiated ITN voucher scheme for pregnant women is part of the Tanzanian commitment to improve access to ITNs for pregnant women as a whole. It is hoped that via mass health education and substantial price subsidy, some of these socio-economic inequities in access will also be addressed.

## Conclusions

The findings highlight the importance of women's knowledge of malaria in pregnancy and of antenatal attendance for the uptake of preventative interventions. Now that effective malaria interventions are available and there is political will to implement them, to maximise the potential for health impact, it is essential to empower the intended recipients of interventions by providing the knowledge which can influence their health decisions.

## Authors' contributions

RN carried out the fieldwork and performed preliminary analysis. CD & HR contributed to study design and logistics. TM performed statistical analysis and supervised the study. All authors read, edited and approved the final manuscript.

**Table 2:** Socio-economic breakdown of population.

		n/293	%
Age	15–19	49	17
	20–29	185	63
	30+	59	20
Gravidity	Primigravidae	124	42
	Mutigravidae	169	58
Residence	Urban	124	42
	Rural	169	58
Education	None	44	15
	Primary	217	74
	Secondary +	32	11
Marital status	Married	207	71
	Unmarried	86	29
Travel time to MCH	< 1 hour	252	86
	1–2 hours	20	7
	>2 hours	21	7
Household ownership	Radio	264	90
	Bicycle	224	76
	TV	16	5
	M/phone	13	4
	Bednet	245	83
Religion	Christian	104	35
	Moslem	189	65
Main source of health information	Community	36	12
	Health workers	83	28
	Media	174	59
Used IPTp-SP	Once	166	57
	Twice	34	12
Used bednet	Yes	207	71
Used ITN	Yes	141	48
Health education MCH	Yes	133	45
Knowledge score	Low	86	29
	Medium	110	38
	High	97	33
